# Obstetrics, Gynecology, Pelvic and Abdominal Surgery

**Published:** 1898-04

**Authors:** Wm. Warren Potter

**Affiliations:** Buffalo. N. Y., President New York State Medical Examining and Licensing Board


### OBSTETRICS, GYNECOLOGY, PELVIC AND ABDOMINAL SURGERY.

Conducted by WM. WARREN POTTER, M. D., Buffalo, N. Y., President New York State Medical Examining and Licensing Board.

### TO PRESERVE THE PERINEUM.

OLIVER (*Am. Med.-Surg. Bulletin*) says: It is my practice to introduce two fingers of the right hand into the vagina and with each pain stretch the perineum in advance of the head;

I have often found extreme rigidity disappear in a few minutes under this treatment. The patient's attention being occupied by the severity of the pain, no objection is ever raised to this procedure. When the head begins to distend the vulva the real work begins, but full expansion has by this time been secured.

Two fingers are introduced behind the occiput and this part of the head is brought well down under the pubic arch. The diameter of the head passing through the outlet will thus be materially lessened and so also will be the tension on the perineum.

Surgically clean cloths, wrung out in hot water, applied directly to a tense perineum often secures good results.

---

### FIBRO-CYSTIC MAMMARY DEGENERATION.

George F. Hulbert, of St. Louis, (*Am. Jour. Surg. and Gynec.*,) reported to the Southern surgical and gynecological association, at its recent meeting, two cases of fibro-cystic degeneration of the female breast, due to the use of the "bust developer." This apparatus produces a partial vacuum in which the mammary gland is drawn repeatedly, producing an intense hyperemia. As a result of this oft-repeated engorgement the fibro-cystic tumors develop. This form of trauma is probably quite common nowadays and may lead to the formation of many such tumors in the near future. The question of import is: Are the highly-developed busts resultant from the use of the "developers" always pathological? And, if so, what will be the effect on future lactation if the breasts be left unoperated?

---

### TAMPONMENT IN POST-PARTUM HEMORRHAGE.

Dr. Marx, (*New York Med. Jour.*, January 28,) in an article entitled Practical measures in obstetrical management, has this to say in regard to post-partum hemorrhage: Quick, precise action is required. No time is to be lost recalling theoretical measures. Means that have stood the test of time must be used, and used at once, to bring on firm and good uterine contractions. I have thrown aside, he says, everything but one or two measures. I employ but one hot intrauterine douche and if this procedure does not bring about the desired result I do not resort to irrelevant and dangerous measures, such as direct compression, ice, persulphate of iron, lemon, vinegar and the like, *ad infinitum*, but proceed to pack the uterus with gauze, toweling or anything I have at hand.

I never go to a case without five yards of gauze being on hand. This is a surgical means of controlling hemorrhage.

The technique of gauze tamponment is simple: one hand over the uterus, while with the other the gauze is passed in, until no more can be introduced. So long as the gauze remains, bleeding cannot occur. The packing can be safely removed at the end of twenty-four hours and renewed if necessary.—*Medical Age.*

---

### PRECOCIOUS MATURITY.

THE *Boston Medical and Surgical Journal* reports the examination of a child one year old. She was fat and healthy; the breasts were enlarged, the nipples not developed; the hair on the pubes was a quarter of an inch long. Townsend observed the process of menstruation; about half a drachm of blood lay on her clothing. The child weighed twenty-eight pounds and was thirty inches in height. At birth she weighed nine pounds; when four months old she cut her first tooth and when examined had seven incisors. The breasts were seen to enlarge at the age of three months and at six months blood issued from the vulva. The mother said the child had menstruated every five weeks.

Mores (*loc. cit.*) observed a similar case; the infant menstruated at nine months and when examined—one year and three months old—was quite regular. Her weight then was thirty-six pounds (at birth it was fourteen pounds); her height was thirty-two and a half inches. The breasts were very well developed, the nipples large with a dark pink areola. The cervix and uterus could be plainly made out on digital examination.

---

### PICRIC ACID DRESSING ON THE UMBILICAL CORD.

ROCHON (*Rev. Obstet. Internat.; Brit. Med. Jour.*) points out that three kinds of dressing are applied to the cord, the oily, the moist and the dry. To the first he objects that it is imperfectly antiseptic, and is opposed to the keratogenic transformation of the young epidermic elements; the second method is sufficiently antiseptic, but it delays the fall of the cord, and often leaves an imperfect cicatrix; while the third, by the rapid desiccation of the cord which it causes, produces the danger of premature separation and hemorrhage. He proposes the following: The cord is surrounded by a piece of absorbent cotton, soaked in a 1-200 solution of picric acid. Thus the decomposition of the cord is prevented and cicatrisation of the umbilicus is aided. A single dressing may suffice, but it is best to repeat it on the second or third day.

